# Advances in understanding the genetic architecture of antibody response to paratuberculosis in sheep by heritability estimate and LDLA mapping analyses and investigation of candidate regions using sequence-based data

**DOI:** 10.1186/s12711-023-00873-4

**Published:** 2024-01-10

**Authors:** Mario Graziano Usai, Sara Casu, Tiziana Sechi, Sotero L. Salaris, Sabrina Miari, Giuliana Mulas, Maria Giovanna Cancedda, Ciriaco Ligios, Antonello Carta

**Affiliations:** 1 Research Unit Genetics and Biotechnology – Agris Sardegna, 07100 Sassari, Italy; 2 Istituto Zooprofilattico Sperimentale Della Sardegna G. Pegreffi, 07100 Sassari, Italy

## Abstract

**Background:**

Paratuberculosis is a contagious and incurable disease that is caused by *Mycobacterium avium* subsp. *paratuberculosis* (MAP) with significant negative effects on animal welfare and farm profitability. Based on a large naturally infected flock over 12 years, we analyzed repeated enzyme-linked immunosorbent assay tests (ELISA), OvineSNP50 BeadChip genotypes and whole-genome sequences imputed from 56 influential animals. The main goals were to estimate the genetic parameters of proxy traits for resistance to MAP, identify genomic regions associated with the host’s immune response against MAP and search for candidate genes and causative mutations through association and functional annotation analyses of polymorphisms identified by sequencing.

**Results:**

Two variables were derived from ELISA tests. The first, a binary variable, assessed the infection status of each animal over the entire productive life, while the second considered the level of antibody recorded over time. Very similar results were obtained for both variables. Heritability estimates of about 0.20 were found and a significant region capturing 18% and 13% of the genetic variance was detected on ovine chromosome 20 by linkage disequilibrium and linkage analysis on OvineSNP50 positions. Functional annotation and association analyses on the imputed sequence polymorphisms that were identified in this region were carried out. No significant variants showed a functional effect on the genes that mapped to this region, most of which belong to the major histocompatibility complex class II (MHC II). However, the conditional analysis led to the identification of two significant polymorphisms that can explain the genetic variance associated with the investigated genomic region.

**Conclusions:**

Our results confirm the involvement of the host’s genetics in susceptibility to MAP in sheep and suggest that selective breeding may be an option to limit the infection. The estimated heritability is moderate with a relevant portion being due to a highly significant region on ovine chromosome 20. The results of the combined use of sequence-based data and functional analyses suggest several genes belonging to the MHC II as the most likely candidates, although no mutations in their coding regions showed a significant association. Nevertheless, information from genotypes of two highly significant polymorphisms in the region can enhance the efficiency of selective breeding programs.

**Supplementary Information:**

The online version contains supplementary material available at 10.1186/s12711-023-00873-4.

## Background

Paratuberculosis (PTB), also known as Johne’s disease, is a contagious disease caused by infection with the gram-positive bacterium *Mycobacterium avium* subsp. *paratuberculosis* (MAP). It affects ruminant species and can manifest as chronic granulomatous enteritis. Paratuberculosis is a very common disease worldwide [1]⁠⁠. Nevertheless, the true prevalence in sheep is very difficult to estimate. Published data on the prevalence suggest a large variability between countries or geographical regions [1–3]⁠⁠⁠. Some studies carried out in Italy reported seroprevalence rates ranging from 66 to 73% at the flock level and from 10 to 15% at the individual level⁠ [3, 4]⁠.

In sheep, the main route of transmission of MAP is faecal-oral. Lambs are usually infected early in life by ingestion of bacteria from the teats or the pasture contaminated by faeces from infected animals [5, 6]. Although MAP is unable to reproduce itself outside of the host cells, it can survive for many months as a spore-like form, so that an environment without animals can remain infectious for a long time [7]. Once infected, animals go through a latency phase that can last several months or years. Then, the disease can remain asymptomatic, with a majority of infected animals being asymptomatic and not developing clinical signs, or can evolve into two main forms (paucibacillary and multibacillary) [8]⁠⁠. Both these forms can result in weight and production loss, emaciation and finally in the death of the infected animals.

Thus, in infected flocks, PTB results in significant negative effects on both animal welfare and farm profitability [9–11]⁠⁠. Moreover, several studies have suggested the implication of MAP in Crohn’s disease in humans [12, 13]⁠⁠. *Mycobacterium avium* subsp. *Paratuberculosis* has also been linked to several human autoimmune diseases, such as type 1 diabetes, rheumatoid arthritis, Hashimoto’s thyroiditis, and multiple sclerosis [14]⁠. Although the causal role of MAP in human diseases has not been proven [15–17]⁠, the possibility of zoonotic transmission remains a significant risk to public health.

There is no effective treatment against MAP infection and the control strategies based on culling seropositive animals are very expensive and have limited effectiveness due to the long period of latency from infection to seroconversion and the lack of sensitive diagnostic tests [18]⁠. Vaccination is known to reduce PTB prevalence, but its effectiveness varies between flocks [19]. Moreover, vaccination is not allowed in some countries because of a possible interference with tuberculosis diagnostic tests.

The most common diagnostic tests used to define individual PTB status are serum ELISA, milk ELISA, faecal culture, and faecal PCR. These tests, although at different extents, are characterized by high specificities and by variable and usually limited sensitivities [18, 20]⁠⁠.

The role of host genetics in susceptibility to MAP infection has been largely studied in cattle. As reviewed by Brito et al. [21], heritability estimates for this trait in this species ranged from 0.03 to 0.57 depending on the diagnostic tests, statistical models and populations [22–30]. Moreover, several genome-wide association studies (GWAS) have been carried out and numerous quantitative trait loci (QTL) have been mapped on nearly all the bovine chromosomes. Indeed, to date, six and 601 QTL associated to “Johne’s disease tolerance” and “*M. paratuberculosis* susceptibility”, respectively, have been listed in the CattleQTLdb (release 5.1). The variability in terms of number and locations of the QTL between different studies could be due to differences in the methods used to define infected and healthy cohorts [29, 31, 32], statistical methodologies (*i.e*., linear [29], threshold models [33], case–control studies [34]), genetic markers (microsatellites [35]; SNPchip: [30, 31, 36–39]; and whole‑genome sequence data [29, 40]). Several positional candidate genes for MAP infection have been subsequently identified or suggested.

Furthermore, several candidate genes have been investigated due to their involvement with susceptibility to other mycobacterial diseases, including human tuberculosis or human leprosy, to their known role in disease pathogenesis or to their links to the susceptibility of humans to Crohn’s disease. As Purdie et al. [41] and Kravitz et al. [42] report in their in-depth reviews on the topic, most of these genes are necessary for innate immune activation, signaling and the subsequent development of adaptive immunity. Thus, polymorphisms in the s*olute carrier family 11 member 1 *gene (*SLC11A1*) [43, 44], *interferon gamma* gene (*INFG*) [43], *nucleotide-binding oligomerization domain* gene (*NOD2*) [45], several interleukin genes and their receptors [46], toll-like receptor genes (*TLR*) [47, 48] and the major histocompatibility complex (*MHC*) genes [49], have been reported to be significantly associated with various PTB phenotypes.

In sheep, the number of studies on the genetic architecture of the resistance/susceptibility to PTB is much smaller. Breed differences in resistance to MAP have been observed in some studies [50, 51]⁠. The reported estimates of heritability range from 0.15 to 0.28 when estimated with threshold models [52, 53] and from 0.07 to 0.24 when estimated for binary trait (survived or died from PTB diagnosed post mortem) [54].

The few studies on candidate genes performed in this species identified significant associations between PTB susceptibility and microsatellite polymorphisms in the *SLC11A1* and *MHC* genes [55] or polymorphisms in the *TLR* genes [56, 57]. To date, only one genomic scan based on the OvineSNP50 BeadChip has been published in sheep. This study has shown suggestive associations between some genes or regions and susceptibility to PTB, but it was based on a small sample of animals (100) and markers (5000 single nucleotide polymorphisms (SNPs)) [58].

In the work reported here, the host’s genetic resistance to PTB in sheep was studied based on data collected on a large experimental flock. Phenotypes consisted of repeated enzyme-linked immunosorbent assay (ELISA) tests performed over time. Ewes were genotyped with the OvineSNP50 BeadChip and whole-genome sequences (WGS) were imputed based on the WGS of 56 influential animals. The main goals were to estimate the genetic parameters of proxy traits for resistance to PTB, identify genomic regions associated with the host’s immune response against MAP, and search for candidate genes and causative mutations through association and functional annotation analyses of polymorphisms identified by WGS data.

## Methods

### Experimental population

The individuals investigated in the present study belong to the female resource population (FRP) of the Sarda breed that is raised on an experimental farm located in the south of Sardinia. The FRP was set in 1999 with the first generation of ewes consisting of 928 back-crosses originating from 10 F_1_ Lacaune × Sarda rams and Sarda pure-breed females. The following generations were obtained by mating adult ewes from FRP with rams from the Sarda Herd Book. The average size of the flock was approximately 900 ewes with a replacement rate of ~ 25%. To date, approximately 5500 ewes have been bred within the FRP. Ewes of the FRP are routinely measured for several production, health and functional traits [59, 60]. The flock is managed following the farming system that is commonly adopted by commercial farms in Sardinia. Feeding is based on grazing natural or cultivated swards supplemented with hay, silage and concentrate. Lambing periods are autumn and late winter for adult ewes and primiparous ewes, respectively. Animals of the nucleus flock are naturally exposed to MAP infection and no control measures, treatment or vaccination against PTB is applied.

In the experimental flock, breeding females derive from adult ewes that lamb between November and December, as a result of artificial insemination or single-sire natural mating that occurs in June and July. Lambing ewes are kept on grassland during the day and housed during the night. After lambing and throughout the suckling period, the ewes are kept together. Lambs follow their mothers outside during grazing and inside during the night within one common indoor pen.

At weaning, at about 30 days of age, female lambs are separated from their mothers and gathered into one common pen, where feeding and water are provided in shared feeders and drinkers. From weaning to their first lambing, the female lambs are kept in a single group separated from the adult ewes: in spring, they start grazing, and in late summer–early autumn, they are naturally mated with adult rams resulting in late winter first lambings.

The experiment on resistance to PTB included 3088 ewes born from 1999 to 2011 from 100 sires. The number and the size of the sire-families varied over years. The first generation born in 1999 derived from 712 Sarda purebred dams and 10 Lacaune × Sarda F_1_ sires with on average 80 daughters each. From 2002 to 2009, five to six sire-families of around 40 ewes each were generated per year. In 2010 and 2011, the number of mating sires increased to 20 per year resulting in an average sire family size of 10 daughters. Ewes born from 2002 to 2011 derived from 90 purebred rams from the Sarda Herd Book mated with 1437 adult ewes from FRP. In each mating season, a specific set of breeding rams was used. Only seven rams needed two mating seasons to achieve the planned number of daughters. Overall, the 3088 ewes derived from 2670 lambings of 2149 dams. The number of daughters bred per lambed ewe was on average 1.16 (1 for the 84% and 2 for the 16% of the lambings). The average number of daughters per dam across parities was 1.4 (1 for the 68%, 2 for the 23% and more than 2 for the 9% of the dams).

### Serological data

**Fig. 1:**
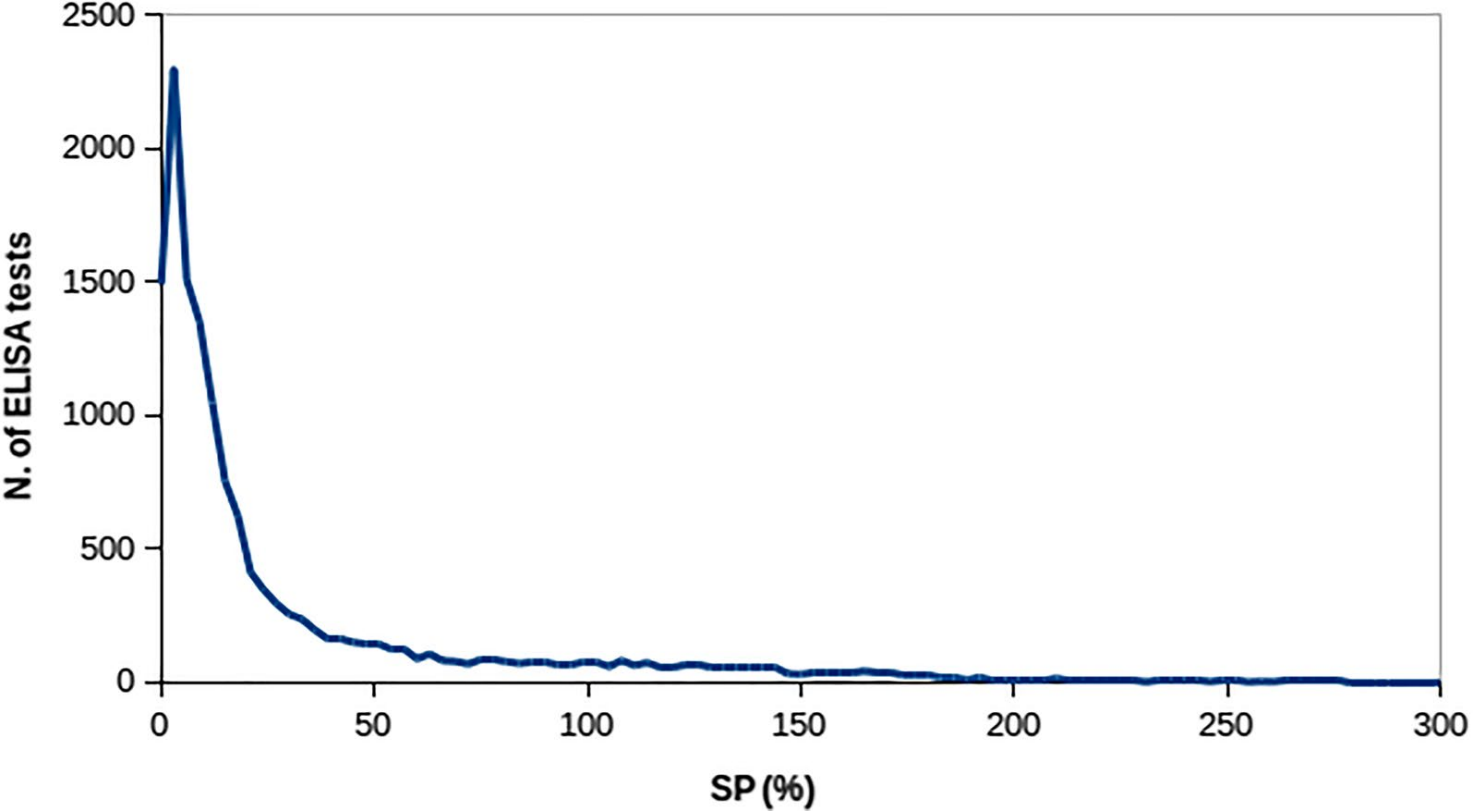
Distribution of sample-to-positive ratio (SP) values

Collection of serological data in the experimental population started in 2001 and ended in 2012. During this period, 3088 ewes born from 1999 to 2011 were blood sampled with intervals between consecutive samplings ranging from 5 to 12 months. The number of blood samples per head was 4.7 on average, ranging from 1 to 8. Overall, 14,482 blood samples were collected (Table 1). Blood samples were tested using the ELISA screening test (Idvet, France or Pourquier ELISA, France) and the test results were expressed on the basis of the percentage of the sample-to-positive ratio (SP) calculated as:

1 $${{\text{SP}}}_{{\text{i}}}=100*\frac{{{\text{OD}}}_{{\text{ij}}}-{\overline{{\text{OD}}} }_{{\text{j}}}^{{\text{neg}}}}{{\overline{{\text{OD}}} }_{{\text{j}}}^{{\text{pos}}}-{\overline{{\text{OD}}} }_{{\text{j}}}^{{\text{neg}}}},$$

where ${{\text{OD}}}_{{\text{ij}}}$ is the optical density of blood sample ${\text{i}}$ on plate ${\text{j}}$; ${\overline{{\text{OD}}} }_{{\text{j}}}^{{\text{neg}}}$ is the optical density average of negative controls of plate ${\text{j}}$, and ${\overline{{\text{OD}}} }_{{\text{j}}}^{{\text{pos}}}$ is the optical density average of positive controls of plate ${\text{j}}$. A test was considered as positive when SP was $\ge$ 70% (Fig. 1).

**Table 1:** Summary of blood samples and serum ELISA tests

Number of samples/heads	Number of head	Number of samples	Average age (months)	Proportion of positives samples
1	160	160	37	0.33
2	411	822	35	0.20
3	213	639	36	0.21
4	1021	4084	42	0.16
5	72	360	29	0.26
6	426	2556	35	0.18
7	419	2933	37	0.12
8	366	2928	36	0.14
Total	3088	14,482	37	0.16

### Molecular data

All 3088 phenotyped ewes, their 100 sires and 10 Sarda grand-sires were genotyped with the Illumina Inc. OvineSNP50 BeadChip (50 K hereafter). Filtering of SNPs was performed by setting the call rate and minor allele frequency thresholds at 95% and 1%, respectively. The ovine genome assembly v4.0 and the SNPchimMpv.3 software [61] were used to construct the genetic map by assuming 1 Mb = 1 cM. Unmapped SNPs and SNPs on sex chromosomes were not included in the study. Finally, 43,390 SNPs were retained for further analyses.

Among the genotyped animals described above, 56 individuals had been previously fully resequenced. A first group of 24 animals (22 ewes and 2 sires) had been chosen within the framework of previous projects and were whole-genome resequenced with a target coverage of 12×. They were selected to mimic trios for opposite alleles at QTL segregating in the Sarda breed for various traits of interest [62]⁠. A second group, consisting of 32 Sarda rams (30 sires and 2 grand-sires), was resequenced more recently with a 30× target coverage. They were selected based on their genetic impact on FRP to maximize the portion of segregating Sarda gametes included in the sequenced sample. Genomic libraries were prepared using the Illumina’s Nextera DNA Flex kit according to the manufacturer instructions. The quality of dual-indexed libraries was assessed using DNA1000 Chip on the Bioanalyzer 2100 (Agilent) and Qubit fluorometric quantification using Qubit dsDNA BR Assay kits (Invitrogen). Dual-indexed 150-bp paired-end sequencing was performed on an Illumina HiSeq3000 instrument at CRS4 (Center For Advanced Studies, Research and Development in Sardinia https://www.crs4.it/) NGS facility. Whole-genome sequence (WGS) data were processed with a pipeline implemented with Snakemake [63] that was developed at CRS4 and available at https://github.com/solida-core. Briefly, adapter sequences were removed from the short reads, then low-quality ends were trimmed, and sequences shorter than 25 bp after trimming were removed with the TrimGalore (v0.4.5) software [64]. The quality of the reads, before and after trimming, was evaluated with the Fastqc (v0.11.5) tool [65]. Trimmed reads were aligned to the *Ovis aries* reference genome v4.0 (https://www.ncbi.nlm.nih.gov/assembly/GCF_000298735.2) using the Burrow-Wheeler Aligner (BWA v0.7.15) program [66]. Alignments were further sorted, converted to a CRAM file and indexed with Samtools (v1.6) [67]. PCR duplicates were detected with the Picard (v2.18.9) tool [68]. After alignment, joint single nucleotide variant calling was performed using the GATK (v4.0.11.0) software [69], according to the GATK Best practices workflow [70]. In order to apply the GATK variant quality score recalibration, first we ran an initial round of SNP calling and only used the top 5% SNPs with the highest quality scores⁠.

### Descriptive variables

The definition of the infected status of an individual based on the antibody levels assessed by the serum ELISA test is questionable. Several studies pointed out that the PTB status based on the SP cutoff has a low sensitivity and high specificity [18]. Thus, a large proportion of the infected animals is expected to be falsely negative, while a true negative is unlikely to result positive. Therefore, to check the overall consistency of the results, we performed the genetic analyses with two different variables.

First, a binary variable (CAR) was defined by assigning the infected status (CAR = 1) to animals showing at least one test during the recording period with an SP $\ge$ 70. However, CAR roughly approximates the status for the animals that have just one test within the recording period (5%) and neglects the test results after obtention of the first positive test, regardless of whether they were all positive or not (as it was the case for 41% of animals which showed at least one negative test after the first positive one).

The second analyzed variable was SP [Eq. (1)] *i.e*. the level of antibody observed over time without explicit definition of the PTB status.

### Analysis of variance components

As far as the sources of environmental variation are concerned, vertical transmission of MAP from dam to offspring through contaminated milk or fecal contamination of the udder [5] may generate a non-genetic covariance between relatives. Thus, including full-sib or dam family random effects in the model would be the best way to avoid a potential overestimation of the genetic variance. However, these effects were not estimable in our study because of either the small litter sizes or the small number of offspring per dam. Moreover, in our study, the dam status at birth was available for 73% of the whole population (2246 ewes). Considering this sample, 71% of infected ewes were from uninfected dams and the prevalence of infected offspring from infected and uninfected dams was 40 and 28%, respectively. This suggests that, in our experimental population, routes of transmission such as suckling from other ewes or between contemporaries make the effect of the dam-to-lamb vertical transmission less relevant than expected.

The lambing season and the management group as well as different biosafety strategies between years could be potential sources of environmental variation of MAP prevalence since animals could be differently exposed to the pathogen’s infection. However, as described above, the strategy of raising animals in a single management group per year as well as the homogeneity of the management strategies across years make it likely that no systematic environmental effect of the year and/or management group affects the PTB status of an animal. Thus, this factor was not included in the statistical model as fixed effect to avoid differences in prevalence between years being incorrectly attributed to environmental sources of variation rather than to the genetic background of the corresponding yearly set of sires and dams.

Finally, the variance component analyses were performed by two variable-specific animal models.

CAR was analyzed with the following single-trait animal model:

2 $$\mathbf{y}=\bf{1}\upmu +\mathbf{Z}\mathbf{g}+\mathbf{e},$$

where $\mathbf{y}$ is the vector of CAR observations (3088); $\mu$ is the overall mean; $\mathbf{g}$ is the vector of genomic breeding values; $\mathbf{e}$ is the vector of random residuals; **1** is a vector of $1$s; and $\mathbf{Z}$ is the incidence matrix relating $\mathbf{y}$ to $\mathbf{g}$.

SP was analyzed with the following single-trait repeatability animal model:

3 $$\mathbf{y}=\bf{1}\upmu +\mathbf{x}{\text{b}}+\mathbf{U}\mathbf{c}+{\mathbf{Wp}}+{\mathbf{Z}\mathbf{g}}+\mathbf{e},$$

where $\mathbf{y}$ is the vector of observations (14,482 for SP); $\mu$ is the overall mean; ${\text{b}}$ is the fixed effect of age at the test; $\mathbf{c}$ is the vector of the 149 random effects of ELISA plates; $\mathbf{p}$ is the vector of the 3088 random animal non genetic effects; $\mathbf{g}$ is the vector of genomic breeding values; $\mathbf{e}$ is the vector of random residuals; $\bf 1$ is a vector of 1s; $\mathbf{x}$ is the vector of ages (in days) at the test date and $\mathbf{U}$, $\mathbf{W}$ and $\mathbf{Z}$ are incidence matrices.

Vectors $\mathbf{g}$ and $\mathbf{e}$ in both Eqs. (2) and (3) and vectors $\mathbf{c}$ and $\mathbf{p}$ in Eq. (3) were assumed to be distributed as $\mathbf{g}\sim N\left({{\mathbf{0}}},{\mathbf{G}}{\upsigma }_{{\text{g}}}^{2}\right)$, $\mathbf{e}\sim N\left({{\mathbf{0}}},{\mathbf{I}}^{\mathbf{e}}{\upsigma }_{{\text{e}}}^{2}\right)$, $\mathbf{c}\sim N\left({{\mathbf{0}}},{\mathbf{I}}^{\mathbf{c}}{\upsigma }_{{\text{c}}}^{2}\right)$ and $\mathbf{p}\sim N\left({{\mathbf{0}}},{\mathbf{I}}^{\mathbf{p}}{\upsigma }_{{\text{p}}}^{2}\right)$, where $\mathbf{G}$ is a genomic relationship matrix calculated by the Van Raden method 1 [71]⁠; ${\mathbf{I}}^{\mathbf{e}}$, ${\mathbf{I}}^{\mathbf{c}}$ and ${\mathbf{I}}^{\mathbf{p}}$ are specific identity matrices; ${\upsigma }_{\text{g}}^{2}$ is the trait genetic variance, ${\upsigma }_{\text{e}}^{2}$ is the trait residual variance, ${\upsigma }_{\text{c}}^{2}$ is the ELISA plate variance and ${\upsigma }_{\text{p}}^{2}$ is the individual non genetic variance. Variance components were estimated by a REML procedure through the airemlf90 software [72]⁠.

For QTL mapping on CAR, the original variable was used, and for QTL mapping on SP, the average performance deviations (APD) were obtained by averaging individual random residuals ($\mathbf{e}$) and summing-up the genetic and non genetic animal effects ($\mathbf{p}+\mathbf{g}$).

### Linkage disequilibrium and linkage analysis and quantitative trait loci variance

A genome scan to detect genomic regions that are associated with resistance to MAP was carried out on 43,390 50 K SNP positions by using the method proposed by Usai et al. [59]⁠. This method uses principal components (PC) to summarize identity-by-descent probabilities (IBD) between gametes of genotyped individuals. Therefore, the paternal and maternal transmitted gametes (named gametes hereafter) of the genotyped individuals were first reconstructed by the linkage disequilibrium multilocus iterative peeling method [73]⁠ using genotype and pedigree information. Then, at each 50 K SNP position ${\text{l}}$, a matrix (${\mathbf{Q}}_{\mathbf{l}}$) allocating IBD probabilities between gametes of phenotyped individuals was built by combining linkage disequilibrium and linkage analyses (LDLA). The IBD between segments of Lacaune origin (inherited from F_1_ sires) and segments of Sarda origin was set to 0. Moreover, a genome-wide IBD matrix (${\mathbf{Q}}_{\mathbf{g}}$) was calculated by averaging all the ${\mathbf{Q}}_{\mathbf{l}}$ to account for the polygenic effects. At this stage, Usai et al. [59]⁠ proposed the use of principal component analysis to summarize the information of ${\mathbf{Q}}_{\mathbf{l}}$ and ${\mathbf{Q}}_{\mathbf{g}}$ to overcome the nonpositive definiteness of ${\mathbf{Q}}_{\mathbf{l}}$ and to limit the computational needs in handling both IBD matrices. The final model does not include random effects other than the residuals and is solved by a weighted least squares method.

At each SNP position ${\text{l}}$, the model was as follows:

4 $${\mathbf{y}}={\mathbf{1}}\upmu +{\mathbf{Z}}^{\mathbf{q}}{\mathbf{V}}_{\mathbf{l}}^{\mathbf{S}}{{\varvec{\upbeta}}}_{\mathbf{l}}^{\mathbf{S}}+{\mathbf{Z}}^{\mathbf{q}}{\mathbf{V}}_{\mathbf{l}}^{\mathbf{L}}{{\varvec{\upbeta}}}_{\mathbf{l}}^{\mathbf{L}}+{\mathbf{Z}}^{\mathbf{q}}{\mathbf{V}}_{\mathbf{g}}{{\varvec{\upalpha}}}_{\mathbf{l}}+{{\varvec{\upvarepsilon}}}_{\mathbf{l}},$$

where $\mathbf{y}$ is the vector of phenotypes (APD of SP or CAR); $\upmu$ is the overall mean; ${\mathbf{V}}_{\mathbf{l}}^{\mathbf{S}}$ and ${\mathbf{V}}_{\mathbf{l}}^{\mathbf{L}}$ are the matrices including the scores of PC extracted from ${\mathbf{Q}}_{\mathbf{l}}$ that explain more than 99% of the variation of IBD probabilities within Sarda and Lacaune, respectively, and ${{\varvec{\upbeta}}}_{\mathbf{l}}^{\mathbf{S}}$ and ${{\varvec{\upbeta}}}_{\mathbf{l}}^{\mathbf{L}}$ are the vectors of the corresponding fixed effects; ${\mathbf{V}}_{\mathbf{g}}$ is the matrix including the scores of PC that explain more than 99% of the variation of the genome-wide IBD probability matrix ${\mathbf{Q}}_{\mathbf{g}}$ and ${{\varvec{\upalpha}}}_{\mathbf{l}}$ is the vector of the corresponding fixed effects estimated at locus ${\text{l}}$; ${\mathbf{Z}}^{\mathbf{q}}$ is an incidence matrix relating phenotypes with gametes and ${{\varvec{\upvarepsilon}}}_{\mathbf{l}}$ is the vector of residuals calculated at locus ${\text{l}}$, assuming that ${\bf{\varepsilon }}\sim {\text{~N}}\left( {\bf{0}{\text{,}}{{\bf{R}}^{ - 1}}{\bf{\sigma }}_{\bf{\varepsilon }}^2} \right)$ with $\mathbf{R}$ being a diagonal matrix with phenotype weight as diagonal elements. For the variable CAR, weights were assumed to be 1 throughout, and thus $\mathbf{R}$ is an identity matrix. For the variable SP, with analyzed phenotypes being APD, to take the different reliability of the individual APD resulting from the different number of ELISA tests per animal into account, the individual weight of the phenotype of the ewe ${\text{i}}$ (${{\text{R}}}_{\text{ii}}$) was calculated as ${{\text{R}}}_{{\text{ii}}}=1- \frac{{\upsigma }_{{\text{e}}}^{2}}{{{\text{n}}}_{{\text{i}}}^{{\text{T}}}\left({\upsigma }_{{\text{g}}}^{2}+{\upsigma }_{{\text{p}}}^{2}\right)}$, where ${{\text{n}}}_{{\text{i}}}^{{\text{T}}}$ is the number of ELISA tests carried out on ewe ${\text{i}}$; ${\upsigma }_{{\text{e}}}^{2}$, ${\upsigma }_{{\text{g}}}^{2}$ and ${\upsigma }_{{\text{p}}}^{2}$ are the estimated variances from the animal model in Eq. (3).

The aim of this analysis was to identify QTL that segregate in the Sarda breed. Thus, at each SNP position ${\text{l}}$, the null hypothesis that the effects of Sarda PC were zero (${{\text{H}}}_{0}:{\upbeta }_{{\text{l}}}^{{\text{S}}}=0$) was tested by an F-test where the sum of the squared residuals of the full model in Eq. (4) was compared with that obtained by the following reduced model:

5 $$\mathbf{y}=\mathbf{1}\upmu +{\mathbf{Z}}^{\mathbf{q}}{\mathbf{V}}_{\mathbf{l}}^{\mathbf{L}}{{\varvec{\upbeta}}}_{\mathbf{l}}^{\mathbf{L}}+{\mathbf{Z}}^{\mathbf{q}}{\mathbf{V}}_{\mathbf{g}}{{\varvec{\upalpha}}}_{\mathbf{l}}+{{\varvec{\upvarepsilon}}}_{\mathbf{l}}^{*}.$$

The genome-wide (GW) significance threshold was determined by the Bonferroni correction of the significance level chosen for the analysis (0.05) for the total number of tested positions (43,390). Thus, the GW significant threshold, in negative logarithm with base 10, was set to 6. This approach is conservative since it does not account for the linkage disequilibrium (LD) between the SNP positions tested. Significant positions identified on the same chromosome were clustered into QTL regions (QTLR) on the basis of the correlations between phenotypes predicted by the QTL effects (${\widehat{\mathbf{y}}}_{\mathbf{Q}\mathbf{l}}={\mathbf{Z}}^{\mathbf{q}}{\mathbf{V}}_{\mathbf{l}}^{\mathbf{S}}{\widehat{{\varvec{\upbeta}}}}_{\mathbf{l}}^{\mathbf{S}}+{\mathbf{Z}}^{\mathbf{q}}{\mathbf{V}}_{\mathbf{l}}^{\mathbf{L}}{\widehat{{\varvec{\upbeta}}}}_{\mathbf{l}}^{\mathbf{L}}$) as proposed by Usai et al. [59]⁠.

Moreover, to appreciate the potential impact of a marker-assisted selection approach, the QTL variance for the most significant position of each QTLR was estimated by including in the animal models in Eqs. (2) and (3) the random effects of the identified QTL. To deal with the nonpositive definite nature of the IBD matrix ${\mathbf{Q}}_{\mathbf{l}}$ calculated at the QTLR peak position ${\text{l}}$, a positive definite submatrix (${\mathbf{Q}}_{\mathbf{l}}^{\mathbf{p}\mathbf{d}}$) was extracted from ${\mathbf{Q}}_{\mathbf{l}}$. To maximize the information captured by ${\mathbf{Q}}_{\mathbf{l}}^{\mathbf{p}\mathbf{d}}$, gametes included in ${\mathbf{Q}}_{\mathbf{l}}^{\mathbf{p}\mathbf{d}}$ were iteratively selected based on their impact on ${\mathbf{Q}}_{\mathbf{l}}$ and the IBD with the previously selected gametes. Then, each gamete carried by a phenotyped ewe was related to the gamete in ${\mathbf{Q}}_{\mathbf{l}}^{\mathbf{p}\mathbf{d}}$ with which it had the highest IBD probability. In our study, this latter probability was on average ~ 0.99 (± 0.05). Thus, the two variable-specific animal models in Eqs. (2) and (3) were updated as follows:

For CAR:

6 $$\mathbf{y}=\mathbf{1}\upmu +\mathbf{Z}\mathbf{g}+{\mathbf{Z}}_{\mathbf{l}}^{\mathbf{p}\mathbf{d}}{\mathbf{q}}_{\mathbf{l}}+\mathbf{e},$$

and for SP:

7 $$\mathbf{y}=\mathbf{1}\upmu +\mathbf{x}{\text{b}}+\mathbf{U}\mathbf{c}+{\mathbf{Wp}}+\mathbf{Z}\mathbf{g}+{\mathbf{Z}}_{\mathbf{l}}^{\mathbf{p}\mathbf{d}}{\mathbf{q}}_{\mathbf{l}}+\mathbf{e},$$

where ${\mathbf{q}}_{\mathbf{l}}$ is a vector of random effects of the selected gametes included in ${\mathbf{Q}}_{\mathbf{l}}^{\mathbf{pd}}$ and assumed to be distributed as ${\mathbf{q}}_{\mathbf{l}}\sim N\left({\mathbf{0}},{\mathbf{Q}}_{\mathbf{l}}^{\mathbf{pd}}{\upsigma }_{{\text{ql}}}^{2}\right)$; ${\upsigma }_{{\text{ql}}}^{2}$ is the variance of the QTL; ${\mathbf{Z}}_{\mathbf{l}}^{\mathbf{p}\mathbf{d}}$ is an incidence matrix relating the paternal (maternal) gamete of the phenotyped ewes with ${\mathbf{Q}}_{\mathbf{l}}^{\mathbf{p}\mathbf{d}}$; the remaining elements of Eqs. (6) and (7) are the same as in Eqs. (2) and (3). Variance components were estimated by a REML procedure through the airemlf90 software [72]⁠.

### Analysis of sequence data

Quantitative trait loci regions as defined above were further investigated using information from WGS data. Biallelic SNPs passing the filtering and falling in these target QTLR were extracted from the assembled sequences of the 56 resequenced animals (WGS SNPs). Then, the parental gametes of the phenotyped ewes were imputed from 50 K data to WGS by the procedure proposed by Usai et al. [74]. The imputation procedure consisted of a first step where the phase of each parental gamete ${\text{k}}$ (where ${\text{k}}=1$ or ${\text{k}}=2$ identify the paternal or maternal transmitted gamete, respectively) carried by the sequenced animal ${\text{j}}$ (${{\text{h}}}_{{\text{jk}}}^{{\text{s}}}$) was iteratively reconstructed by estimating the probability of carrying the reference ${\text{P}}\left({{\text{h}}}_{{\text{jkl}}}^{{\text{s}}}={\text{R}}\right)$ and the alternative ${\text{P}}\left({{\text{h}}}_{{\text{jkl}}}^{{\text{s}}}={\text{A}}\right)$ allele at each WGS SNP position ${\text{l}}$. These probabilities were conditioned to the genotypic information from sequencing and the IBD between gametes of sequenced individuals at the neighboring 50 K SNP positions.

Then, at each WGS SNP position ${\text{l}}$, the probabilities that gamete ${\text{k}}$ of the phenotyped ewe ${\text{i}}$ (${{\text{h}}}_{{\text{ik}}}^{{\text{p}}}$) carried the reference ${\text{P}}\left({{\text{h}}}_{{\text{ikl}}}^{{\text{p}}}={\text{R}}\right)$ and the alternative ${\text{P}}\left({{\text{h}}}_{{\text{ikl}}}^{{\text{p}}}={\text{A}}\right)$ allele were inferred on the basis of the gametic phases of sequenced animals and the IBD between gametes of the sequenced animals with gametes of the phenotyped ewes. The information content of each imputed gamete (${{\text{w}}}_{{\text{ikl}}}$) was defined by the general formula ${{\text{w}}}_{{\text{ikl}}}=\frac{{{\text{n}}}_{{\text{a}}}{\sum }_{{\text{m}}=1}^{{\text{m}}={\text{na}}}{\text{P}}{\left({{\text{h}}}_{{\text{ikl}}}^{{\text{p}}}={\text{m}}\right)}^{2}-1}{{{\text{n}}}_{{\text{a}}}-1}$, which for biallelic cases (${{\text{n}}}_{{\text{a}}}=2$) can be simplified to ${{\text{w}}}_{{\text{ikl}}}={\left[{\text{P}}\left({{\text{h}}}_{{\text{ikl}}}^{{\text{p}}}={\text{R}}\right)-{\text{P}}\left({{\text{h}}}_{{\text{ikl}}}^{{\text{p}}}={\text{A}}\right)\right]}^{2}$.

Two approaches were used to assess the accuracy of imputation in the target regions. The first approach considered only the sequenced animals and all the WGS SNPs. The accuracy was defined by 56 leave-one-out cross-validations and corresponded to the correlation between the true and the imputed genotypes. The second approach considered all the phenotyped ewes and only 50 K SNP positions. In this case, the accuracy was calculated as the correlation between ${\text{P}}\left({{\text{h}}}_{{\text{ikl}}}^{{\text{p}}}={\text{R}}\right)$ and the actual occurrence of the same allele defined in the LDLA analysis on 50 K data.

Then, an association analysis was run in the target regions by regressing the investigated variables on the allele dosage of the reference allele (R). The model tested at each WGS SNP position ${\text{l}}$ was the following:

8 $$\mathbf{y}=\mathbf{1}\upmu +{\mathbf{p}}_{\mathbf{l}}{\upgamma }_{\mathbf{l}}+{\mathbf{Z}\mathbf{V}}_{\mathbf{g}}{{\varvec{\upalpha}}}_{\mathbf{l}}+{{\varvec{\upvarepsilon}}}_{\mathbf{l}},$$

where the terms $\mathbf{y}$, $\mathbf{1}\upmu$, ${\mathbf{ZV}}_{\mathbf{g}}{{\varvec{\upalpha}}}_{\mathbf{l}}$ and ${{\varvec{\upvarepsilon}}}_{\mathbf{l}}$ are the same as for the LDLA mapping model in Eq. (4); ${\upgamma }_{{\text{l}}}$ is the additive substitution effect of R and ${\mathbf{p}}_{\mathbf{l}}$ is the vector allocating the dosage of allele R of the phenotyped ewes. To calculate the allele dosage, the probability ${\text{P}}\left({{\text{h}}}_{{\text{ikl}}}^{{\text{p}}}={\text{R}}\right)$ from the imputation was weighted for its information content ${{\text{w}}}_{{\text{ikl}}}$, to limit the impact on the regression of gametes imputed with low precision. Therefore, the weighted probability of each gamete ${{\text{i}}}_{{\text{k}}}$ carrying R at WGS SNP position ${\text{l}}$ (${{\text{P}}}^{{\text{w}}}\left({{\text{h}}}_{{\text{ikl}}}^{{\text{p}}}={\text{R}}\right)$) was calculated as ${{\text{P}}}^{{\text{w}}}\left({{\text{h}}}_{{\text{ikl}}}^{{\text{p}}}={\text{R}}\right)={{\text{w}}}_{{\text{ikl}}}\left[{\text{P}}\left({{\text{h}}}_{{\text{ikl}}}^{{\text{p}}}={\text{R}}\right)-{{\text{f}}}_{{\text{l}}}^{{\text{R}}}\right]+{{\text{f}}}_{{\text{l}}}^{{\text{R}}}$, where ${{\text{f}}}_{{\text{l}}}^{{\text{R}}}$ is the allele frequency of the allele R at SNP ${\text{l}}$ calculated as ${{\text{f}}}_{{\text{l}}}^{{\text{R}}}=\sum {{\text{w}}}_{{\text{ikl}}}{\text{P}}\left({{\text{h}}}_{{\text{ikl}}}^{{\text{p}}}={\text{R}}\right)/\sum {{\text{w}}}_{{\text{ikl}}}$ and the individual allele dosage was calculated as ${{\text{p}}}_{{\text{il}}}={{\text{P}}}^{{\text{w}}}\left({{\text{h}}}_{{\text{i}}1{\text{l}}}^{{\text{p}}}={\text{R}}\right)+{{\text{P}}}^{{\text{w}}}\left({{\text{h}}}_{{\text{i}}2{\text{l}}}^{{\text{p}}}={\text{R}}\right)$, where subscripts “$1$” and “$2$” indicate the paternal and maternal gametes inherited by the phenotyped ewe ${\text{i}}$.

The null hypothesis that the effect of SNP l was zero (${{\text{H}}}_{0}:{\upgamma }_{{\text{l}}}=0$) was tested by an F-test.

Functional annotation of the WGS SNPs included in each QTLR was performed by using the NCBI 4.0 sheep genome annotation release 102 and the SnpEff software v4.3.t [75]⁠. Genes included in the most significantly associated regions, annotated by orthology to human genes from the OrthoDB v10 database, were analyzed using the KEGG and gene ontology (GO) biological process terms databases with the web-based software WebGestalt [76].

Moreover, a conditional analysis [77] was carried out to determine if the multiple significant WGS SNP effects detected in a QTLR were due to LD or to captured independent effects. The conditional association analysis was performed by a step-wise procedure. At each step, the association model in Eq. (8) was updated by adding, as fixed effect, the most significant WGS SNP; then all the remaining WGS SNPs were retested with the updated model and the new p-values were used to select additional SNPs to be added in the model as fixed effect. The procedure continued until the most significant WGS SNP did not exceed the significance threshold of $-{{\text{log}}}_{10}\left({\text{P}}-{\text{value}}\right)$ = 6.

## Results

### Serological data

**Fig. 2:**
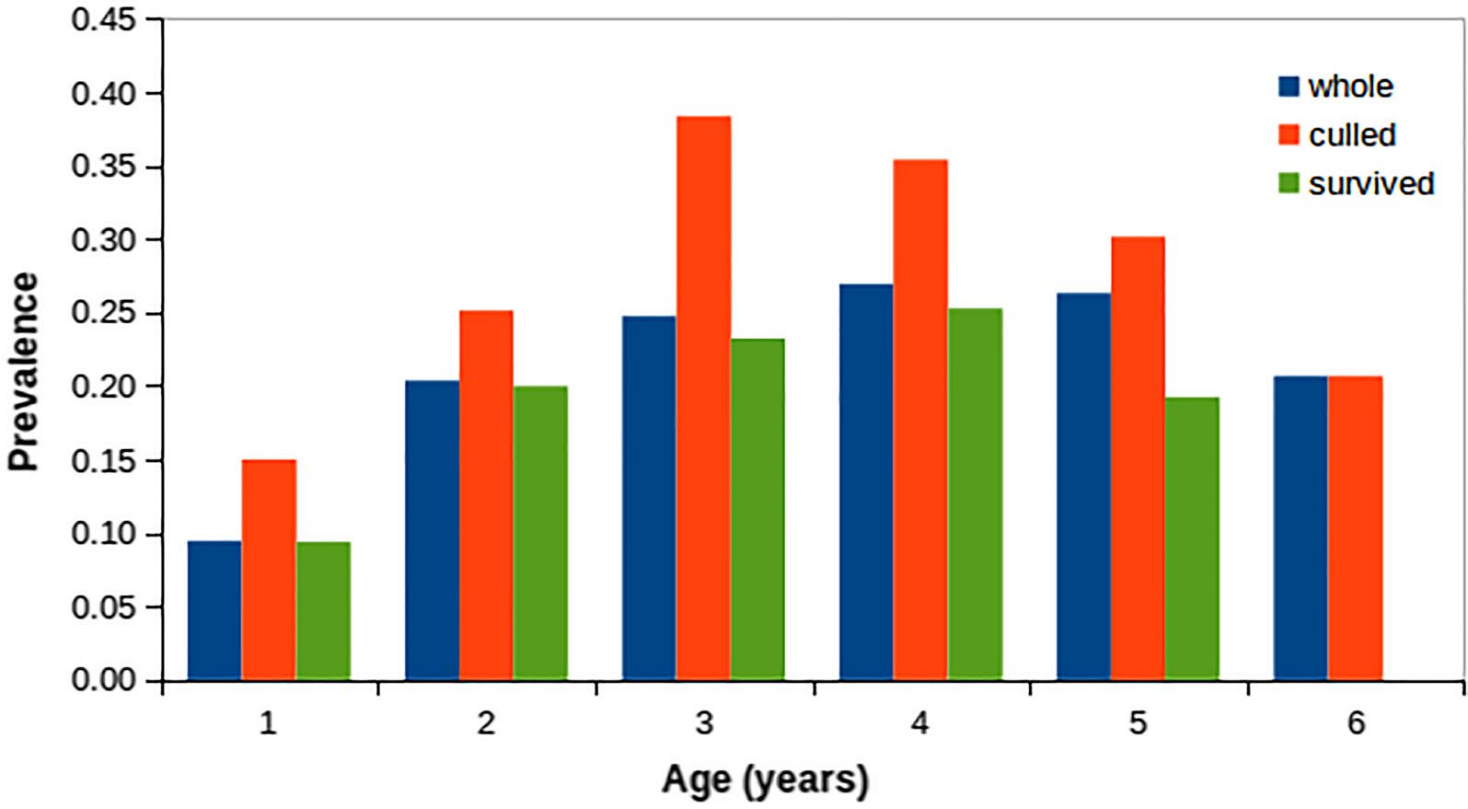
Prevalence of infected animals per age class. Whole: prevalence in the entire set of contemporaries; culled: prevalence in the portion of contemporaries died or removed; survived: prevalence in the portion of contemporaries alive in the next year of age

Approximately 16% of the 14,482 ELISA tests were positive (SP > 70%) and 29.7% of the 3088 investigated ewes were considered infected since they had at least one positive test during the recording period. Figure 2 shows the prevalence of infected animals (CAR = 1) observed in FRP for different age classes. Prevalence increases until 4 years of age reaching a maximum of approximately 27%; then it decreases in older ewes. Since animals are usually infected early in life, the progressive increase in infection prevalence in the first four age classes is probably caused by differences in the length of the infection-to-seroconversion period. The decrease in infection prevalence in 5 to 6 year-old animals is probably due to the greater chance of the infected animals to be voluntary or involuntary culled earlier in life with respect to healthy animals. In fact, Fig. 2 shows that the infection prevalence in the group of culled animals at each age class is always higher than that observed in surviving animals of the same age, especially for 3 and 4 year-old ewes. Thus, although no selective culling based on serological status was applied, the mortality due to PTB combined with the voluntary culling not clearly attributable to PTB, significantly reduced the prevalence of infection in older animals. During the recording period, 115 ewes died within six months after the last ELISA test with clinical signs of PTB and 77% of them were also classified as infected (CAR = 1).

Figure 1 shows the distribution of the observed SP values. The overall mean was 33 (± 47) and the most frequently observed value was 2. The skewed distribution of this variable is mainly due to the large variability of SP values in positive tests. In several studies, SP has been log-transformed to make the data more concordant with the normal distribution. In this study, we present results from non-transformed SP since no relevant differences between log-transformed and original SP were observed both in terms of genetic parameters’ estimates and QTL mapping.

### Variance components

Table 2 shows the estimates of variance components obtained with the two variable-specific animal models. The heritability estimates were 0.20 for both CAR and SP. The repeatability estimate for SP was 0.68. The variance explained by the random effect of the ELISA plate was 2.9% (± 0.41) of the total SP variance, indicating the good stability of the ELISA results in our study.

**Table 2:** Estimates and standard errors (SE) of ELISA plate variance (${\widehat{\upsigma }}_{{\text{c}}}^{2}$), individual non genetic variance (${\widehat{\upsigma }}_{{\text{p}}}^{2}$), genetic variance (${\widehat{\upsigma }}_{{\text{g}}}^{2}$), residual variance (${\widehat{\upsigma }}_{{\text{e}}}^{2}$), total variance (${\widehat{\upsigma }}_{{\text{tot}}}^{2}$), repeatability ($\widehat{{\text{r}}}$) and heritability ($\widehat{{{\text{h}}}^{2}}$) for CAR and SP

Parameter	CAR (SE)	SP (SE)
${\widehat{\upsigma }}_{{\text{c}}}^{2}$		69 (10)
${\widehat{\upsigma }}_{{\text{p}}}^{2}$	–	1079 (49)
${\widehat{\upsigma }}_{{\text{g}}}^{2}$	0.0427 (0.007)	453 (60)
${\widehat{\upsigma }}_{{\text{e}}}^{2}$	0.1676 (0.006)	736 (10)
${\widehat{\upsigma }}_{{\text{tot}}}^{2}$	0.2102 (0.006)	2268 (51)
$\widehat{{\text{r}}}$	–	0.68 (0.008)
$\widehat{{{\text{h}}}^{2}}$	0.20 (0.029)	0.20 (0.024)

### Linkage disequilibrium and linkage analysis and quantitative trait loci variance

**Fig. 3:**
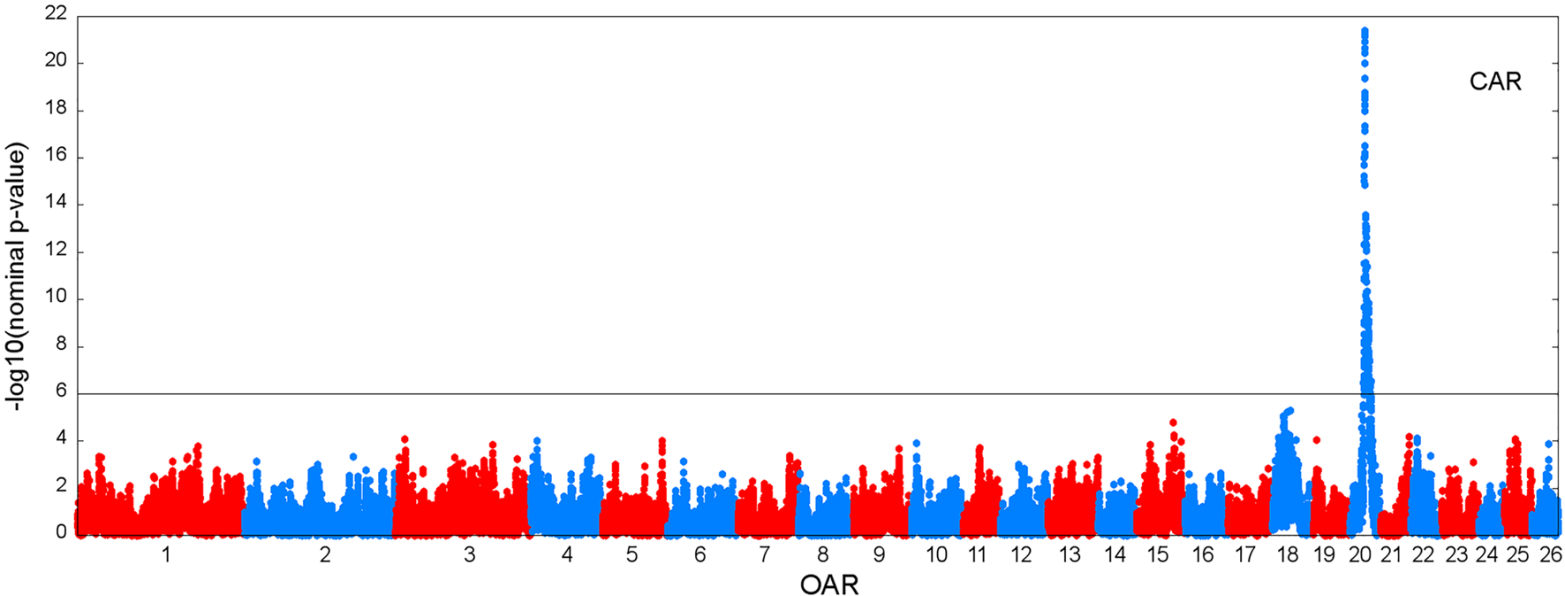
Manhattan plot of the − log_10_(nominal P-values) against the 43,390 positions of the 50 K SNPs obtained by LDLA mapping that was carried out based on CAR. The grey line indicates the 0.05 genome-wide significance threshold determined by Bonferroni correction for 43,390 tests

**Fig. 4:**
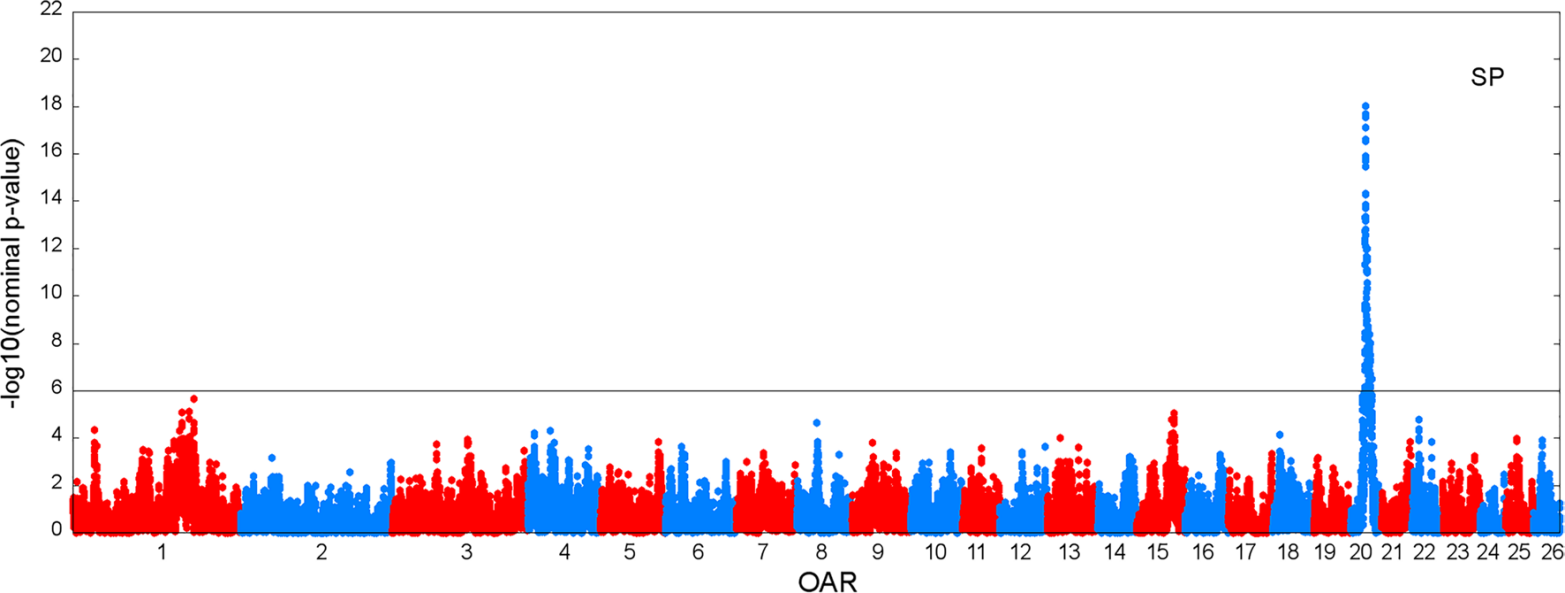
Manhattan plot of the − log_10_(nominal P-values) against the 43,390 positions of the 50 K SNPs obtained by LDLA mapping that was carried out based on SP. The grey line indicates the 0.05 genome-wide significance threshold determined by Bonferroni correction for 43,390 tests

Figures 3 and 4 show the results of the genome scan that was carried out on the 43,390 positions of the 50 K SNPs by LDLA mapping. The significance profiles obtained for CAR (Fig. 3) and SP (Fig. 4) were very similar even if more significant associations were obtained with CAR. One hundred and twelve SNP positions exceeded the 0.05 GW significance threshold ($-{{\text{log}}}_{10}\left({\text{P}}-{\text{value}}\right)=6$) for both variables. Forty-four and four specific significant locations, were identified for CAR and SP, respectively. All the significant positions were located on *Ovis aries* chromosome (OAR) 20 within a region between 23 and 35 Mb. The most significant position for the two variables corresponds to two close SNPs located at ~ 24.8 Mb (Table 3). For both variables, significant SNPs were clustered in a unique QTLR since the phenotypes predicted by the QTL (${\widehat{{\text{y}}}}_{{\text{Ql}}}$) at each significant SNP position were strongly correlated (r > 0.45) to the phenotypes predicted by the QTL at the position of the peak. Additional positions close to the GW significance threshold were detected on OAR15 at ~ 62 Mb for both variables; on OAR18 at ~ 30 Mb for CAR and on OAR1 at ~ 200 Mb for SP.

**Table 3:** QTL regions from the LDLA analysis

Variable	OAR	Significant SNPs (n)	Range (Mb)	Highest peak		
				SNP name	Position (bp)	$-{{\text{log}}}_{10}\left({\text{Pvalue}}\right)$
SP	20	116	23.49–35.13	rs419287784	24,791,184	18.025
CAR	20	156	23.12–35.13	rs413015672	24,860,179	21.396

OAR: *Ovis aries* chromosome; significant SNPs: number of 50 K SNPs exceeding the 0.05 genome-wide significance threshold ($-{{\text{log}}}_{10}\left({\text{Pvalue}}\right)$ > 6); range: position of the first and last significant SNP of the QTL region; highest peak: the most significant SNP 50 K position

The Q–Q plot of P-values are reported in Additional file 1: Figs. S1 and S2 and nominal P-values and false discovery rates are in Additional file 2: Tables S1 and S2 for all the 50 K SNP positions.

Concerning the estimation of QTL variance, the positive definite sub-matrices (${\mathbf{Q}}_{\mathbf{l}}^{\mathbf{p}\mathbf{d}}$) extracted from the whole IBD matrices (${\mathbf{Q}}_{\mathbf{l}}$) calculated at QTLR peak positions on OAR20, included 52 and 56 gametes for CAR and SP, respectively, and captured 0.99 (± 0.05) of the ${\mathbf{Q}}_{\mathbf{l}}$ information for both variables. Table 4 shows the REML estimates of the variance components of the animal models including the random QTL effects. The variance explained by the QTL was ~ 3.6% and 2.5% of the total phenotypic variance and ~ 18% and 13% of the cumulative genetic variance (${\widehat{\upsigma }}_{{\text{g}}}^{2}+{\widehat{\upsigma }}_{{\text{q}}}^{2}$) for CAR and SP, respectively. The higher proportion of variance explained by the QTL based on CAR with respect to that based on SP could explain the higher significance values obtained with the variable CAR. Moreover, for both of the investigated variables, the sums of the QTL and polygenic variances (${\widehat{\upsigma }}_{{\text{g}}}^{2}+{\widehat{\upsigma }}_{{\text{q}}}^{2}$) as well as their ratios with the total variances $\left({\widehat{\upsigma }}_{{\text{g}}}^{2}+{\widehat{\upsigma }}_{{\text{q}}}^{2}\right)/{\widehat{\upsigma }}_{{\text{tot}}}^{2}$ (Table 4) corresponded well to the genetic variances and heritabilities estimated with the animal models without QTL (Table 2).

**Table 4:** Estimates and standard errors (SE) of ELISA plate variance (${\widehat{\upsigma }}_{{\text{c}}}^{2}$), individual non genetic variance (${\widehat{\upsigma }}_{{\text{p}}}^{2}$), genetic variance (${\widehat{\upsigma }}_{{\text{g}}}^{2}$), QTL variance (${\widehat{\upsigma }}_{{\text{q}}}^{2}$), residual variance (${\widehat{\upsigma }}_{{\text{e}}}^{2}$), total variance (${\widehat{\upsigma }}_{{\text{tot}}}^{2}$) estimated for CAR and SP when random QTL effects were included in the animal models

Parameter	CAR (SE)	SP (SE)
${\widehat{\upsigma }}_{{\text{c}}}^{2}$		68 (10)
${\widehat{\upsigma }}_{{\text{p}}}^{2}$	–	1058 (47)
${\widehat{\upsigma }}_{{\text{g}}}^{2}$	0.0334 (0.006)	379 (55)
${\widehat{\upsigma }}_{{\text{q}}}^{2}$	0.0074 (0.002)	56 (13)
${\widehat{\upsigma }}_{{\text{e}}}^{2}$	0.1653 (0.006)	737 (10)
${\widehat{\upsigma }}_{{\text{tot}}}^{2}$	0.2063 (0.006)	2230 (47)
${\widehat{\upsigma }}_{{\text{g}}}^{2}+{\widehat{\upsigma }}_{{\text{q}}}^{2}$	0.0408 (0.006)	436 (56)
$\left({\widehat{\upsigma }}_{{\text{g}}}^{2}+{\widehat{\upsigma }}_{{\text{q}}}^{2}\right)/{\widehat{\upsigma }}_{{\text{tot}}}^{2}$	0.20 (0.027)	0.20 (0.023)
${\widehat{\upsigma }}_{{\text{q}}}^{2}/{\widehat{\upsigma }}_{{\text{tot}}}^{2}$	0.036 (0.008)	0.025 (0.006)
${\widehat{\upsigma }}_{{\text{q}}}^{2}/\left({\widehat{\upsigma }}_{{\text{g}}}^{2}+{\widehat{\upsigma }}_{{\text{q}}}^{2}\right)$	0.18 (0.046)	0.13 (0.032)

### Analysis of sequence data

According to the QTLR identified by LDLA mapping (Table 3), the genomic region between 23.1 and 35.2 Mb on OAR20 was further investigated by using WGS data. Within this region, 167,895 biallelic WGS SNPs were polymorphic in the 56 sequenced animals and were imputed to the phenotyped individuals by exploiting the IBD probability at the 191 50 K SNP positions that were estimated for the LDLA mapping. The average information content of the phenotyped ewes across WGS SNPs was 0.90 (± 0.06) with a strong difference between gametes of Lacaune or Sarda origin. In fact, the average information content was 0.98 (± 0.002) and 0.33 (± 0.13) for Sarda and Lacaune gametes, respectively. This result was expected since sequenced animals were selected with the aim of representing most of the Sarda variability regardless of the Lacaune gametes that were, consequently, poorly represented. In fact, in this specific region, only 2.6% of the genomic information from sequenced animals was of Lacaune origin. This percentage was too low to accurately impute gametes of Lacaune origin, which represent ~ 12% of the gametes of the phenotyped ewes. For this reason, we set the information content of all the Lacaune gametes to zero when the association analysis was performed on WGS SNPs.

The accuracy of imputation calculated by cross-validation on sequenced animals was on average 0.93 (± 0.04), ranging from 0.84 to 0.98. The accuracy of imputation estimated at the 191 50 K positions on phenotyped ewes was on average higher than 0.99 (± 0.01) for all the Sarda gametes.

**Fig. 5:**
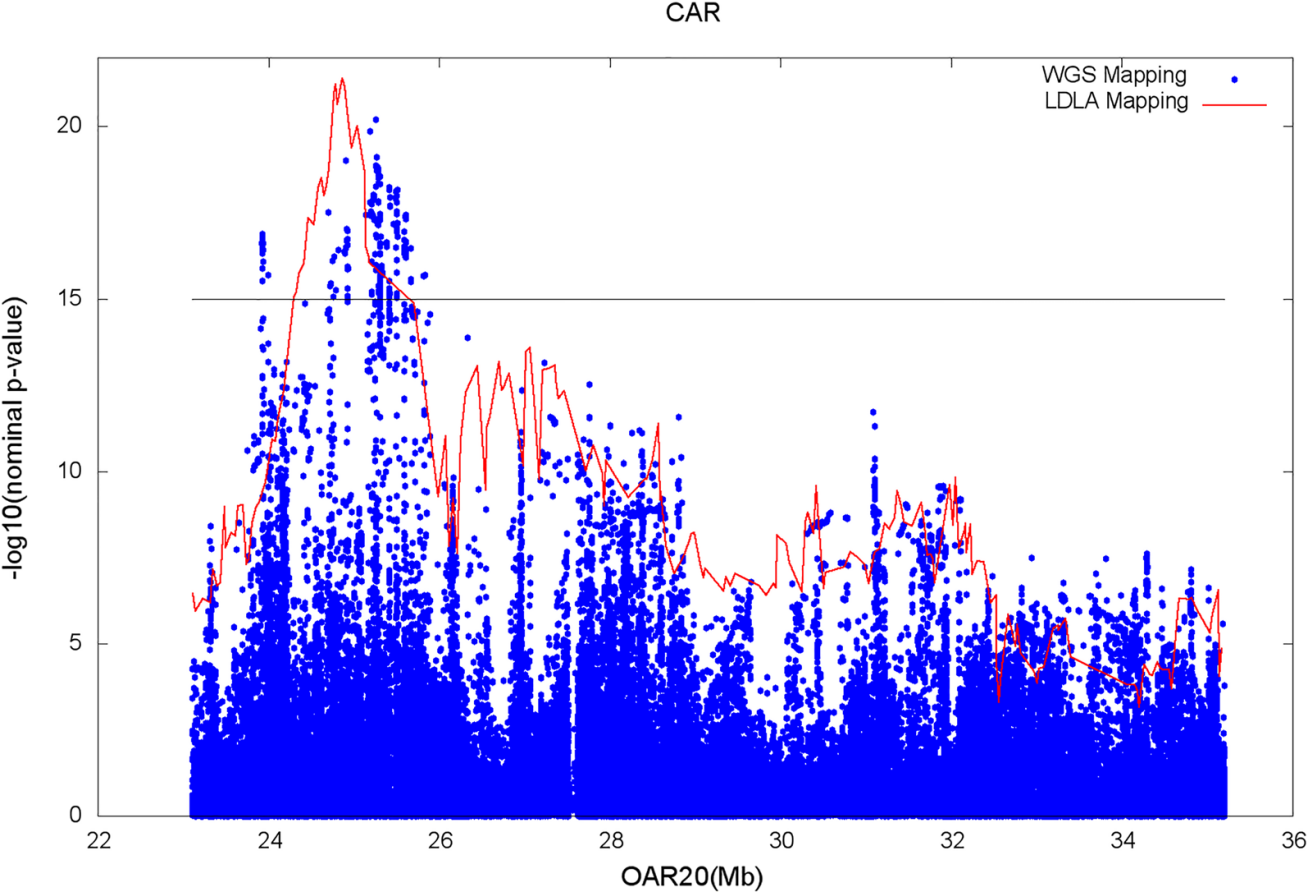
Statistic tests profile of the LDLA mapping (red line) and Manhattan plot of the association analysis on WGS SNPs (blue dots) for CAR. The black line indicates the upper quarter of the peak significance threshold for the WGS association analysis

**Fig. 6:**
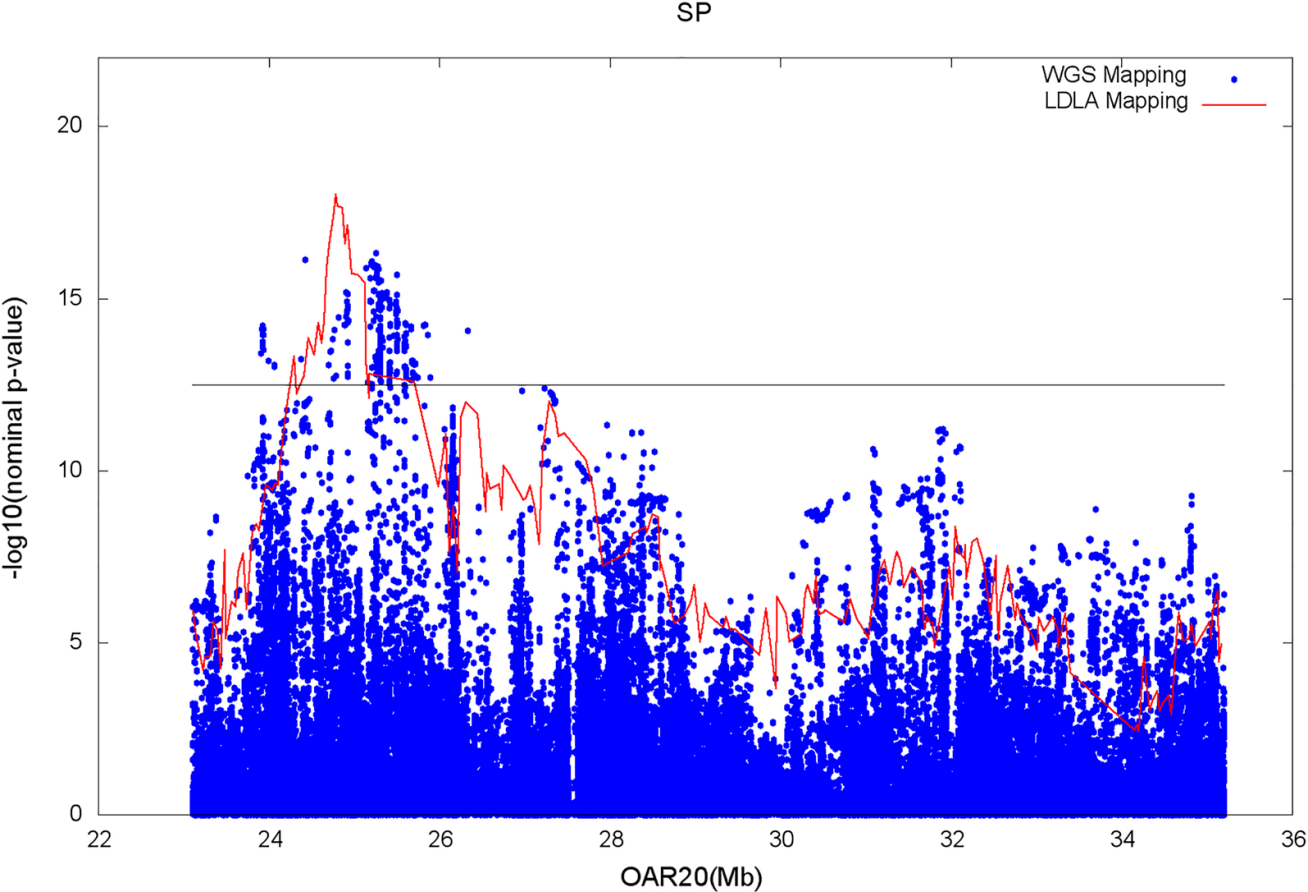
Statistic tests profile of the LDLA mapping (red line) and Manhattan plot of the association analysis on WGS SNPs (blue dots) for SP. The black line indicates the upper quarter of the peak significance threshold for the WGS association analysis

Manhattan plots from the association analysis on the WGS SNPs included in the QTLR identified by LDLA mapping, are in Figs. 5 and 6 for CAR and SP, respectively. In agreement with the results of LDLA mapping, the profiles obtained for the two investigated variables follow the same pattern, although CAR outperformed SP in terms of P-value. For both variables, the most significant WGS SNP was detected at 25,253,298 bp (rs400535267) with a $-{{\text{log}}}_{10}\left({\text{P}}-{\text{value}}\right)$ of 20.2 and 16.3 for CAR and SP, respectively. Moreover, considering the top 500 significant SNPs (corresponding to a $-{{\text{log}}}_{10}\left({\text{P}}-{\text{value}}\right)$ higher than 14.4 and 12.6 for CAR and SP, respectively), 455 SNPs (91%) were in common and the correlation between $-{{\text{log}}}_{10}\left({\text{P}}-{\text{value}}\right)$ was 0.78.

The 50 K SNPs included in the region showed low significance levels when compared with the most significant WGS SNPs. Indeed, the highest peaks showed a $-{{\text{log}}}_{10}\left({\text{P}}-{\text{value}}\right)$ equal to 10.3 and 8.9 for CAR and SP, respectively. Given the overall similarity of the results and in order not to overload the following sections, only the CAR variable will be considered hereafter.

The analysis of the functional annotation focused on the most significantly associated region, which consisted of the interval between 23.77 and 25.83 Mb and was bounded by the extreme positions of WGS SNPs that were included in the upper quarter of the peak ($-{{\text{log}}}_{10}\left({\text{P}}-{\text{value}}\right)$>15). This interval included 35,506 SNPs and harbored 31 protein coding genes. The number of transcripts per gene reported in the NCBI 4.0 sheep genome annotation release 102 was on average 2.4, ranging from 1 to 15. SNPeff provided 167,562 effects in the region. The enrichment analysis was possible for 28 genes for which 23 orthologous human genes were identified. The top ranked biological functions were all related to the immune response process: GO:0050852: T-cell receptor signaling pathway; GO:0060333: interferon-gamma-mediated signaling pathway and GO:0019886: antigen processing and presentation of exogenous peptide antigen via MHC class II. Moreover, the top ranked KEGG pathway was the inflammatory bowel disease, a term that includes human chronic gastrointestinal inflammatory disorders such as ulcerative colitis and Crohn’s disease. Thirteen genes enriched this pathway, two of which encode interleukins (*IL17F* and *IL17A*) and 11 encode major histocompatibility complex class II (MHC II) molecules (*DQA, DQB, LOC101108696, LOC101109220, LOC101109492, LOC101109747, LOC101119856, LOC105603927, OVAR-DRB1, OVAR-DRB3 and LOC101120871*). These 11 genes also enriched the three most represented GO processes.

As far as the functional annotation is concerned, Table 5 reports a summary of the annotation impacts and effects of the investigated SNPs on OAR20. Nine SNPs were predicted to have a HIGH impact on multiple transcripts of the *PKHD1*, *LOC101106976* (*glutathione S-transferase A4-like*), *DQA*, *BTNL2* and *LOC101110277 (butyrophilin-like protein 1*) genes. However, none of these variants was significantly associated with the CAR variable ($-{{\text{log}}}_{10}\left({\text{P}}-{\text{value}}\right)$ <5). A MODERATE impact was predicted for 356 missense variants spread over 28 of the 31 identified genes. The most significant SNP ($-{{\text{log}}}_{10}\left({\text{P}}-{\text{value}}\right)$=14.8) of this impact category was located at 25,591,715 bp (rs1089342381) corresponding to exon 2 of the *DQA* gene (HGVS.p = p.Thr49Ile). Furthermore, three missense variants with suggestive significance $-{{\text{log}}}_{10}\left({\text{P}}-{\text{value}}\right)$>10) were identified in the *DQA*, *PKHD1* and *BTNL2* genes.

**Table 5:** Annotation impacts and effects of WGS SNPs located within the interval between 23.77 and 25.83 Mb on OAR 20

Annotation impact	Annotation effect	n SNP	n effects	$-{{\text{log}}}_{10}\left({\text{Pvalue}}\right)$	n SNP > 15
High	Stop gained	5	26	4.8	0
	Splice donor variant	2	4	1.4	0
	Splice acceptor variant	1	13	1.1	0
	Stop lost	1	8	0.3	0
Moderate	Missense variant	356	2042	14.8	0
Low	Synonymous variant	324	1849	17.5	3
	Splice region variant	80	342	8.8	0
	Initiator codon variant	1	1	4.0	0
	5′ UTR premature start codon gain variant	47	89	9.3	0
Modifier	Intergenic region	17,333	17,333	20.2	314
	Upstream gene variant	4017	7537	19.9	74
	Downstream gene variant	3255	6202	19.0	27
	Intron variant	16,791	130,561	19.0	65
	Non coding transcript exon variant	214	214	13.8	0
	5′ UTR variant	250	483	10.3	0
	3′ UTR variant	422	1209	7.8	0

nSNP: number of WGS SNPs in the region; n effects: number of corresponding effects; -log_10_(P-value) negative logarithm of the P-value of the most significant SNP; n SNP > 15: number of WGS SNPs exceeding the significance threshold $-{{\text{log}}}_{10}\left({\text{Pvalue}}\right)>15$

Moreover, among the 26 and 12 missense variants identified in the *DQA* and *DQB* genes, only 4 (2 per gene) were predicted to have a probably damaging effect by the PolyPhen-2 tool [78]. However, all of them showed a very low significance level ($-{{\text{log}}}_{10}\left({\text{P}}-{\text{value}}\right)$ < 3).

Concerning SNPs with a predicted impact LOW, 444 variants were identified in the investigated region, which were mostly annotated as synonymous variants. The three most significant LOW impact SNPs ($-{{\text{log}}}_{10}\left({\text{P}}-{\text{value}}\right)$ from 15.2 to 17.5) were located at positions 25,497,603 bp (rs429603683) and 25,497,202 bp (rs1093423097) corresponding to exons 4 and 3 of the *LOC101109492* (*SLA class II histocompatibility antigen, DQ haplotype D alpha chain*) gene and at 25,462,223 bp in the first exon of the *DQB* gene. Six additional synonymous variants with $-{{\text{log}}}_{10}\left({\text{P}}-{\text{value}}\right)$>10 were identified, in the *PKHD1* (3 variants), *PAQR8*, *MCM3* and *OVAR-DRB3* genes.

With the exception of the three synonymous variants with a LOW impact described above, all the 384 significant SNPs included in the upper quarter of the peak ($-{{\text{log}}}_{10}\left({\text{P}}-{\text{value}}\right)>$ 15) were classified as MODIFIER, thus with no impact on the transcripts (Table 6). They were annotated as intron variants (65) or intergenic regions (316). Intron variants were spread across nine of the 31 investigated genes, with the majority (31) falling into introns of the *PKHD1* gene, consistent with the large size of this gene. Another 12 highly significant intronic variants were found in the *LOC101106976* gene and included the most significant intronic SNP. This variant (rs400071669) was located within the fifth intron at 24,906,858 bp and its significance ($-{{\text{log}}}_{10}\left({\text{P}}-{\text{value}}\right)$=19.02) was the fourth highest detected among all the WGS SNPs analyzed. The remaining 22 highly significant SNPs were detected in intronic regions of the *LOC101109492* (8), *OVAR-DRB1* (4), *DQA* (4), *LOC101119856* (3), *TMEM14A* (1), *LOC101107232* (1) and *ELOVL5* (1) genes.

**Table 6:** Functional annotation of WGS SNPs belonging to the upper quarter of the peak [$-{\mathbf{l}\mathbf{o}\mathbf{g}}_{10}\left(\mathbf{P}\mathbf{v}\mathbf{a}\mathbf{l}\mathbf{u}\mathbf{e}\right)>15$] for CAR

Functional annotation	Gene/region	Interval (bp)	n SNP	$-{{\text{log}}}_{10}\left({\text{Pvalue}}\right)$
Synonymous	DQB*	25,457,637–25,462,339	1	17.2
	LOC101109492*	25,492,374–25,497,723	2	17.5
Intron	*PKHD1*	23,775,497–24,227,486	31	16.9
	*TMEM14A*	24,776,903–24,790,779	1	15.2
	*LOC101106976*	24,901,975–24,923,227	12	19.0
	*LOC101107232*	24,929,447–24,941,686	1	15.9
	*ELOVL5*	25,120,790–25,145,587	1	17.4
	OVAR-DRB1*	25,318,507–25,332,926	4	16.8
	LOC101119856*	25,379,045–25,387,727	3	16.6
	LOC101109492*	25,492,374–25,497,723	8	18.1
	DQA*	25,589,973–25,595,946	4	16.5
Intergenic	*TRAM2-TMEM14A*	24,642,641–24,776,902	8	17.5
	*GSTA1-1-LOC101106291*	24,807,779–24,824,652	1	16.4
	*LOC101106720-LOC101106976*	24,874,489–24,901,974	2	16.4
	*LOC101106976-LOC101107232*	24,923,228–24,929,446	2	15.9
	ELOVL5-LOC101108696*	25,145,588–25,279,802	75	20.2
	LOC101108696*-OVAR-DRB1*	25,283,274–25,318,506	128	18.8
	OVAR-DRB1*-LOC101119856*	25,332,927–25,379,044	3	16.6
	LOC105603927*-LOC101109220*	25,412,660–25,431,004	22	18.2
	LOC101109492*-OVAR-DRB3*	25,497,724–25,523,968	31	18.2
	OVAR-DRB3*-DQA*	25,538,379–25,589,972	7	16.9
	DQA*-LOC101120871*	25,595,947–25,654,933	32	17.4
	LOC101120871*-LOC101109747*	25,660,584–25,693,040	3	16.5
	*LOC101110277—LOC101121635*	25,755,893–25,849,941	2	15.7
Total		23,773,103–25,828,335	384	20.2

^*^Major histocompatibility complex class II genes; nSNP: number of WGS SNPs exceeding the significance threshold $-{{\text{log}}}_{10}\left({\text{Pvalue}}\right)>15$; $-{{\text{log}}}_{10}\left({\text{Pvalue}}\right)$ negative logarithm of the p-value of the most significant SNP

The 316 intergenic variants with a $-{{\text{log}}}_{10}\left({\text{P}}-{\text{value}}\right)$>15 were located within 13 intergenic regions delimited by 20 protein coding genes (Table 6). The highest density of SNPs was identified in the region bounded by the *LOC101108696* (*SLA class II histocompatibility antigen, DQ haplotype D alpha chain-like*) and *OVAR-DRB1* genes. However, the most significant SNP detected in this region was ranked only 20th for significance. The most significant SNPs were detected in the region bounded by the *ELOVL5* and *LOC101108696* genes, which are localized just before the intergenic region described above. This region included 18 of the top 20 significant SNPs, including the most significant one. In fact, the most significant variant ($-{{\text{log}}}_{10}\left({\text{P}}-{\text{value}}\right)$= 20.2) was detected at 25,253,298 bp, 107,710 bp from the *ELOVL5* gene and 26,504 bp from *LOC101108696*. This SNP (rs400535267) consisted of a G > T change. The frequency of the reference allele (G) was 0.88 and the additive substitution effect estimated on CAR was -0.223, thus the expected explained variance was 0.0105.

**Fig. 7:**
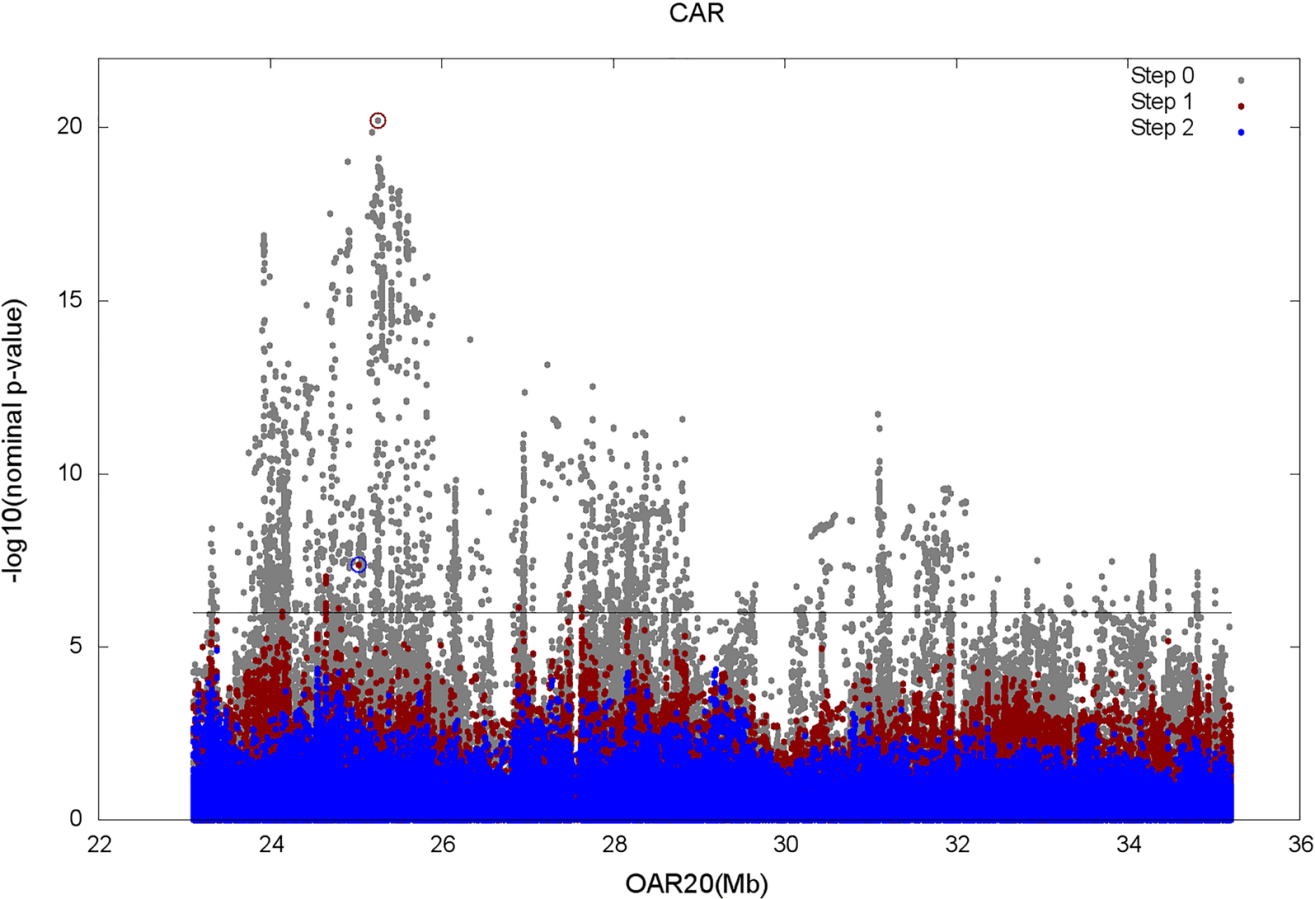
Conditional analysis on WGS SNPs for CAR. Step0: original WGS mapping; Step1: WGS mapping conditioned to SNP rs400535267 (red circle); Step2: WGS mapping conditioned to SNPs rs400535267 and rs605280267 (red and blue circles)

In addition to the rs400535267 SNP, the conditional analysis carried out on all the WGS SNPs in the QTLR for CAR, led to the selection of one more WGS SNP (rs605280267) (Fig. 7), which is located at 25,033,899 bp, within the fifth intron of the *glial cells missing transcription factor 1* (*GCM1*) gene and consisted of an A > C change. The significance of this SNP, estimated by the updated model, was 7.4 $-{{\text{log}}}_{10}\left({\text{P}}-{\text{value}}\right)$; the frequency of the reference allele (A) was 0.86 and the LD with the rs400535267 variant was very low (r^2^ = 0.005). The significance of these two variants tested jointly was 25.6 $-{{\text{log}}}_{10}\left({\text{P}}-{\text{value}}\right)$ and the estimated effects were -0.244 and -0.114 for rs400535267 and rs605280267, respectively.

## Discussion

The analysis of the prevalence of PTB in our population confirmed the relevant negative effect that MAP infection may have on sheep farms’ economy. In fact, we observed that infected ewes have a risk of being culled earlier in life about 50% higher than that of uninfected ones. This increase in risk reaches 90% at 4 years of age (Fig. 2).

In spite of the different nature of the two analyzed variables, the results clearly overlap in terms of heritability estimates and detection of QTL. Overall, the heritabilities of the serological response to PTB estimated based on the genomic relationship matrix were moderate and consistent with previous studies on sheep that used pedigree-based relationship matrices [52–54]⁠⁠. Even if our estimates may be slightly overestimated due the impossibility of estimating the shared maternal environment effect of dam or litter and to the statistical confounding between the genetic background of the dams and their serological status at lambing, our results allow to conclude that selective breeding based on progeny testing and large-scale ELISA tests in the selected population may be an option to enhance the resistance of sheep to MAP infection. The main limitation to the application of such a breeding program is the high cost of collecting the phenotypes. However, some reduction in the number of serological tests needed to assess the infection status of an individual can be achieved by avoiding further tests after the first positive one. For instance, in the present experiment, this approach would have reduced the number of serological tests by 16%.

On the one hand, the significant region on OAR20 identified by LDLA analysis, maps close to a region that affects the resistance to gastro‐intestinal nematodes detected in the same experimental population [79]⁠. Moreover, the orthologous region on bovine chromosome 23 has been found to be significantly associated with resistance to PTB in most of the recent GWAS performed with high-density SNP genotypes or imputed whole-genome sequences [29, 30, 39, 40, 80, 81]. In our population, this region explains a relevant portion of the genetic variance for both variables and could be exploited in selection schemes. However, the direct use of effects estimated by the LDLA approach in a large-scale selective program would be constrained by the difficulty of estimating accurate IBD matrices in populations that are not structured as the current experimental one.

On the other hand, the analysis of this QTLR based on imputed WGS data, confirmed the strong association of many WGS SNP genotypes with resistance to PTB. Moreover, the functional annotation analysis of the most significant segment on OAR20 (approximately between 23.8 and 25.8 Mb), led to the identification of 31 protein coding genes as positional candidates. Gene ontology (GO) and pathway enrichment analyses confirmed that many of these genes are involved in biological processes that are compatible with MAP infection. In particular, 11 genes in this region belong to the MHC class II genes and have a role in both the innate and adaptive immune systems. They encode for molecules involved in the presentation of antigens to T-cells to induce the expression of interferon gamma, which leads to the activation of macrophages and the induction of inflammation. MHC II genes are mainly expressed by the phagocytes and they have been shown to be downregulated in response to MAP infection [82]. Polymorphisms within the MHC II genes also influence the activation of the adaptive immune system, since MHC II molecules loaded with antigens interact with CD4^+^ T cells to induce the antigen-specific immune response [42]. An association between MHC II alleles and susceptibility to MAP has already been suggested in sheep [55] and differential expression of MHC class II genes has been observed in experimentally-infected [83]⁠ or sub-clinically affected [46] cows. Mutations that modify the expression of these genes or the ability to activate T cells have a systemic effect on the host, causing an inappropriate immune response that could change the course of the infection [42]⁠. Although Gossner et al. [84]⁠ did not find evidence of differential expression of MHC II genes in the ileocaecal lymph node of ewes with paucibacillary or multibacillary PTB, Purdie et al*.* [85]⁠ found a differential expression of the *MHC II DQα* and *β* genes in resilient sheep (i.e. animals that had received an infectious dose of MAP and did not develop an infection).

Nevertheless, the joint analysis of the results of WGS mapping and functional annotation of WGS SNPs did not reveal any mutations that had an effect on amino acid sequence and were significantly associated with the antibody response to PTB. In fact, most of the highly significant variants were located outside of the coding regions, especially, in intergenic regions bounded by MHC II genes (Table 6). Among these intergenic regions, the most significant one was located just upstream of the *LOC101108696* (*SLA class II histocompatibility antigen, DQ haplotype D alpha chain-like*) gene. For this gene, Purdie et al. [85]⁠ observed an up-regulated expression in resilient individuals. Although in the annotation release that we used, no functions are associated with highly significant variants, some functional meaning might be revealed by further advances in the annotation of the sheep genome. In addition, although not directly involved in detectable functional effects, the most significant WGS SNP (rs400535267) shows an important statistical effect. The conditional analysis led to the identification of another WGS SNP (rs605280267) that is localized about 200 kb away and may explain the residual portion of genetic variance attributable to the investigated genome segment. It cannot be excluded that the detected effect of this additional WGS SNP could be due to the inability of the top SNP to capture the overall amount of variation at this locus, because of the lack of information content [86]⁠. This additional SNP is within an intronic region of the *GCM1* gene, which does not appear to be involved in biological processes compatible with antibody response to PTB. Overall, the results suggest that information from genotypes at these two WGS SNPs can be exploited to increase the efficiency of selective breeding programs. The possibility that the effects of these significant SNPs may be caused by their LD with other genome variants such as insertions or deletions, copy number variants, or the presence of multiple copies of the *DQA* and *DQB* genes [87, 88] was not investigated in this study since this would have entailed the application of specific bioinformatic analyses and ad hoc statistical models.

Several studies underlined the need of integrating traditional genetic models with epidemiological data for traits based on infectious diseases and demonstrated that quantitative genetic approaches do not account for the feed-back dynamics in the transmission when predicting the response to genetic selection for disease resistance [89–91]. In fact, it has been demonstrated that the response to selection for resistance to infectious diseases is substantially larger than expected for non-contagious traits [89, 92] and that it increases as the prevalence decreases [93]. Taken together, this evidence strengthens the prospects to significantly reduce PTB prevalence by selective breeding for resistance to PTB in sheep populations.

## Conclusions

This study produced significant advances in the understanding of the genetic architecture of the antibody response to paratuberculosis in sheep. The overall results allow us to conclude that the genetic background underlying the host’s resistance to paratuberculosis infection is complex. The estimates of heritability with polygenic models confirm that progeny testing for selective breeding based on large-scale recording of the infection status is a feasible approach. Linkage disequilibrium and linkage analysis detected a highly significant region on ovine chromosome 20. The association analysis with whole-genome sequence data allowed us to markedly reduce the length of the genome segment to be investigated by functional studies depending on the progress of the ovine genome annotation. Moreover, a plausible list of candidate genes in the major histocompatibility complex class II was produced. Information on genotypes at the two identified whole-genome sequence SNPs can enhance the efficiency of selective breeding programs. Further analyses are needed either to detect single nucleotide causative mutations based on updates of the ovine genome assembly and annotation or to investigate the functional role of genome variants such as insertions or deletions and copy number variants.

### Supplementary Information

**Additional file 1: Figure S1.** Quantile–quantile (Q–Q) plot corresponding to the LDLA mapping that was carried out based on CAR. The plot contains the observed − log_10_(p-value) obtained by the LDLA analysis (y axis) plotted against the expected − log_10_(p-value) (x axis). **Figure S2.** Quantile–quantile (Q–Q) plot corresponding to the LDLA mapping that was carried out based on SP. The plot contains the observed − log_10_(p-value) obtained by the LDLA analysis (y axis) plotted against the expected − log_10_(P-value) (x axis).

**Additional file 2: Table S1.** LDLA mapping results for all the 43,390 50 K SNP positions tested for CAR. The table reports the results of the genome scan carried out on the 43,390 positions of the 50 K by LDLA mapping, significances are reported as − log_10_ of both nominal P-values of F-tests and false discovery rate. OAR: *Ovis aries* chromosomes; SNP name and position (bp) name and position in base pair of the 50 K SNP (from the Ovine Genome Assembly v4.0); − log_10_(P-value): negative logarithm of the P-value corresponding to F-test; -log_10_(FDR): negative logarithm of the false discovery rate. **Table S2.** LDLA mapping results for all the 43,390 50 K SNP positions tested for SP. The table reports the results of the genome scan carried out on the 43,390 positions of the 50 K by LDLA mapping, significances are reported as -log_10_ of both nominal P-values of F-tests and false discovery rate. OAR: *Ovis aries* chromosomes; SNP name and position (bp) name and position in base pair of the 50 K SNP (from the Ovine Genome Assembly v4.0); − log_10_(P-value): negative logarithm of the P-value corresponding to F-test; − log_10_(FDR): negative logarithm of the false discovery rate

## Data Availability

The data that support the findings of this study are available from Centro Regionale di Programmazione (CRP), Regione Autonoma della Sardegna but restrictions apply to the availability of these data, which were used under license for the current study, and thus are not publicly available. However, data are available from the authors upon reasonable request and with permission of Centro Regionale di Programmazione (CRP), Regione Autonoma della Sardegna.
