# Supplementary figures and images for: Advances in understanding the genetic architecture of antibody response to paratuberculosis in sheep by heritability estimate and LDLA mapping analyses and investigation of candidate regions using sequence-based data
Source: Genet Sel Evol. 2024 Jan 10;56:5. doi: 10.1186/s12711-023-00873-4 (PMC10777618; doi:10.1186/s12711-023-00873-4)

Figure S1


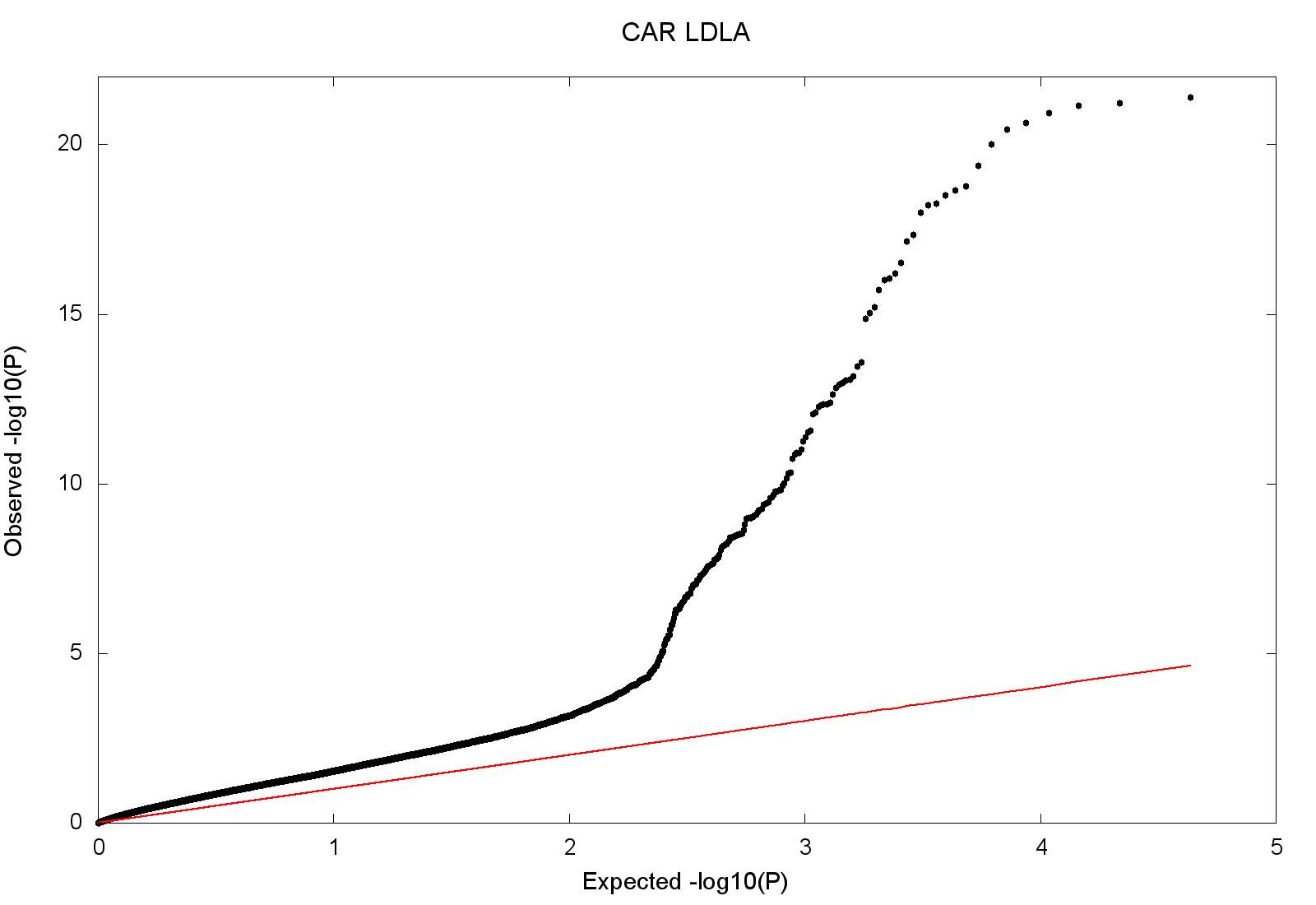


Figure S2


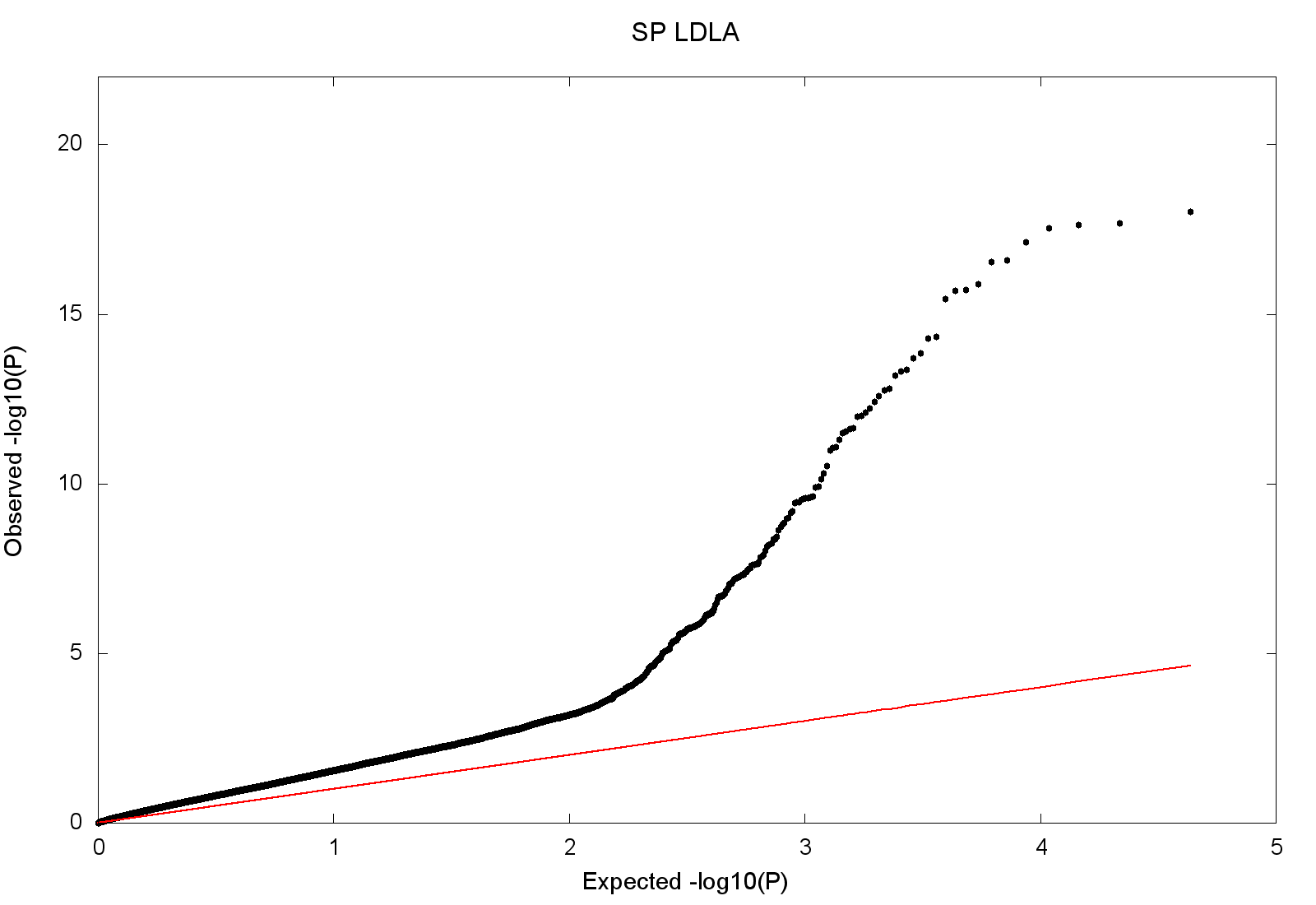

Supplement: Supplementary file 1 — Additional file 1: Figure S1. Quantile–quantile (Q–Q) plot corresponding to the LDLA mapping that was carried out based on CAR. The plot contains the observed − log10(p-value) obtained by the LDLA analysis (y axis) plotted against the expected − log10(p-value) (x axis). Figure S2. Quantile–quantile (Q–Q) plot corresponding to the LDLA mapping that was carried out based on SP. The plot contains the observed − log10(p-value) obtained by the LDLA analysis (y axis) plotted against the expected − log10(P-value) (x axis). [file 12711_2023_873_MOESM1_ESM.docx]
